# Reducing Food Poverty and Vulnerability among the Rural Elderly with Chronic Diseases: The Role of the New Rural Pension Scheme in China

**DOI:** 10.3390/ijerph15061253

**Published:** 2018-06-13

**Authors:** Zhaohua Zhang, Yuxi Luo, Derrick Robinson

**Affiliations:** 1 College of Economics and Management, Shandong Agricultural University, Tai’an 271018, China; 2 School of Economics and Management, Guangxi Normal University, Guilin 541004, China; yluogxnu@mailbox.gxnu.edu.cn; 3 Division of Agriculture and Natural Resources, University of California, San Diego, CA 92123, USA; dearobinson@ucanr.edu

**Keywords:** New Rural Pension Scheme, food poverty, vulnerability, chronic diseases, elderly

## Abstract

Vulnerability to food poverty is the probability of an individual falling below the food poverty line in the near future, which provides a forward-looking welfare analysis. Applying a nationally representative survey dataset, this study investigates the role of the New Rural Pension Scheme (NRPS) in reducing food poverty and vulnerability among the rural elderly with chronic diseases. By designing province-specific food poverty lines to account for variations in the elderly’s needs, as well as the prices across provinces using a least-cost linear programming approach, the food poverty incidences among the elderly with chronic diseases are calculated. Applying a three-stage feasible generalized least squares (FGLS) procedure, the vulnerability to food poverty is estimated. Our results show that food poverty incidence and vulnerability of the elderly with chronic diseases in rural China is 41.9% and 35% respectively, which is 8% and 6% higher, respectively, than the elderly that are in good health. To address the potential endogeneity of pension payment, a fuzzy regression discontinuity (RD) regression is employed to investigate the effects of pension income on food poverty and vulnerability for different population groups. We found that pension income decreases the probability of being food poor and the vulnerability to food poverty among the elderly with chronic diseases by 12.9% and 16.8% respectively, while it has no significant effect on the elderly in good health.

## 1. Introduction

With the largest elderly population in the world, China is now experiencing an unprecedented aging process. By the end of 2015, the population aged 60 and over in China had reached 220 million, accounting for 16% of the total population (Source: National Bureau of Statistics of the People’s Republic of China). It is also projected that, by 2050, this number will exceed 490 million (36% of the population) (Source: Population Division of the Department of Economics and Social Affairs of the United Nations, *Word Population Prospects: the 2015 revision*). Unlike the elderly in developed countries, whose consumption expenditures are largely financed by a pension system, most of the people at an old age in China, especially in rural China, are financed by savings and by transfers from children. However, the implementation of the one-child policy in the last three decades has dramatically increased the dependency ratio at a relatively low income level [1], making the old population face significant uncertainty in their future consumption. As China ages, the elderly experience high incidence of poverty and the deprivation of basic services. To provide income security for old people, social pension programs have been widely regarded and implemented as an important policy tool [2]. With the majority of the elderly living in rural areas, in late 2009, the Chinese government launched the New Rural Pension Scheme (NRPS)—a departure from the traditional family support—to ensure the well-being of the aging older population in rural China. The implementation of the NRPS brought rural China into a new period of social pension systems, which have far-reaching well-being implication for rural residents.

Aging has been an issue for both developed and developing countries, but this phenomenon is more recent in developing countries, and programs to provide support for old people in less developed countries are limited [3]. The NRPS is one of the social pension programs that has been introduced by the Chinese government, targeting the elderly population residing in rural areas. In light of the importance of social pension programs on dealing with aging issues, whether these programs reach their expected goals has triggered the interests of many researchers. A large body of literature has examined the impact of social pension programs on the elderly population. However, considerable attention has been paid to the effects of a social pension program on labor supply [3,4,5], intergenerational economic transfer from adult children to their parents [6,7], and living arrangements [8,9,10]. The channels through which pension income contributes to food poverty and vulnerability among the rural elderly, especially the elderly with chronic diseases, it is not well understood. Food poverty can generally be defined as a situation where a household or an individual lacks the resources to acquire a nutritionally adequate diet [11]. People of an old age often face particular challenges in terms of the financial resources that are required to access foods, as aging imposes a decrement in productivity [12].To maintain caloric intake, the elderly who worry that they will not have adequate money for food reduce the variety in their diet and concentrate their intake on a few low-cost, energy-dense, and nutritionally-poor foods [13]. Major risk factors for the onset of some of the most prevalent chronic diseases are the consumption of diets rich in empty calories and poor in nutrients (e.g., vitamins and phytochemicals) [14]. Therefore, the elderly that are food poor are more likely to experience chronic diseases. Furthermore, suffering from the symptoms of these chronic diseases reduces the elderly’s participation in the labor market, which further reduces their income. Since income is a significant factor in determining whether elders get nutritionally adequate food, old adults with chronic diseases are supposed to be at a higher risk of vulnerability to food poverty, leading to unequal distribution of food poverty incidence and vulnerability between the elderly with chronic diseases and those in good health. Given the challenges of ensuring food security and prevent age-associated chronic diseases so as to promote healthy aging, the Chinese government launched the NRPS targeting rural residents in 2009. This paper has two objectives, it (1) investigates the magnitude and causes of ex-post food poverty and ex-ante vulnerability of the rural elderly with and without chronic diseases, and (2) estimates how this pension reform affects food poverty and vulnerability of the rural elderly with chronic diseases, and tests whether the effects are different for different population groups. Analyzing these questions is challenging for at least two reasons. Firstly, the consumption decision is largely affected by life-cycle patterns and cohort heterogeneity, resulting in difficulties in separating the effect of pension income from age or cohort heterogeneity [5]. Taking advantage of the policy design that rural residents are eligible for pension income beginning at age 60, we apply a fuzzy regression discontinuity (RD) design to overcome this issue. The fuzzy RD approach is often used when the take-up of the pension is incomplete or does not coincide exactly with age eligibility, which is the case in our study, where pension cannot be paid to the elderly immediately after their 60th birthday. The second challenge is the determination of the food poverty line. The elderly’s vulnerability to food poverty is conceptualized as the probability that the elderly will fall below the food poverty line; hence, it is critical to determine the food poverty line in order to analyze the vulnerability. In the absence of data on the calorie consumption of individuals, the direct calorie intake method and food energy intake approach could not be applied. To solve this problem, a least-cost approach, based on a linear programming technique, is applied. By setting nutrition constraints in terms of calories, the least-cost approach selected the food list with the least cost that satisfied the requirements of a healthy diet. This study is different from previous studies, which directly use the uniform official Chinese poverty line, by designing province-specific food poverty lines to account for variations in the elderly’s needs and the prices across provinces. These province-specific food poverty lines make the analysis of food poverty and vulnerability highly robust. 

Focusing on a sample of rural residents aged 50–70, drawn from the China Health and Retirement Longitudinal Survey (CHARLS), our results reveal several key findings. Firstly, food poverty incidence and vulnerability are different between the elderly with chronic diseases and those without any chronic diseases in rural China—the food poverty incidence and vulnerability of the elderly with chronic disease is 8% and 6% higher, respectively, than the elderly in good health. Furthermore, we find that the pension income decreases the probability of being food poor and the vulnerability to food poverty among the elderly with chronic diseases by 12.9% and 16.8% respectively, while it has no significant effect on the elderly in good health. These results are robust for alternative specifications of the adult equivalence of the per day calorie constraint. The rest of this paper is structured as follows. Section 2 provides materials and methods, while discussions of key results are presented in Section 3. Robustness checks and conclusions are provided in Section 4 and Section 5, respectively.

## 2. Materials and Methods

### 2.1. The New Rural Pension Scheme in Rural China

Before 2009, the well-established pension programs in China exclusively covered the elderly population in urban areas, but rural residents, as primarily self-employed agricultural workers, had no safety net prior to the rollout of the NRPS [15]. While urban residents enjoyed a series of social benefits, such as unemployment insurance, health care, and pension, the elderly rural residents were mainly financed by their own savings and by transfers from children. In 2015, the ratio of the population aged 65+ per 100 population, aged 15–64, reached 13%. This ratio was projected to increase to 46.7% in 2050 (Source: Population Division of the Department of Economics and Social Affairs of the United Nations, *Word Population Prospects: the 2015 revision*). The high level of the old-age dependency ratio suggested the high pressure of traditional family support, which limited the financial resources that were available for intergenerational transfers. Furthermore, because of the imbalanced economic development between the urban and rural areas, the per capita income of the urban residents was almost three times that of the rural residents (China Statistical Yearbook, 2016). Although the rural saving rate had increased during the last few decades, there were still families with zero or negative savings, indicating income levels that could not fully cover their consumption expenditures [16]. To ensure income security and the well-being of the rural elderly, China initiated government transfer or social pension programs for the uncovered elderly in rural areas.

The pilot social pension program aimed at rural elderly was introduced at the beginning of 1990s under the supervision of the Ministry of Civil Affairs as an institutional framework for administering a pension program for voluntary-contribution, defined-contribution, and fully funded individual accounts [17]. However, this program placed financial responsibility mainly on individual contributions, and was supplemented by the local collectives, with the central government providing policy support if needed. Because of this financial arrangement, the participation rate of this program was low, and the central government terminated the program in 1999 [18]. In September 2009, China launched the NRPS for rural residents, beginning with 320 pilot counties and covering almost all 2853 counties by 2012 [19].

The NRPS was different from the pilot rural pension program in several aspects. Firstly, according to the NRPS, all of the rural residents aged 16+ who were not enrolled in the urban basic pension program could voluntarily participate in the NRPS, and the pension-eligibility was age determined. The NRPS required participants aged 45 and below at the time when this program was launched, to contribute for at least 15 years in order to be eligible for pension payment upon reaching the age of 60. However, there was no minimum years of contribution that were placed on the participants who were between the ages of 45 and 60 [1]. Secondly, there were three sources of contributions to the pension fund, including an individual premium, with both local and central government subsidies. For the personal contribution, individuals could make a decision about the level of premium among five basic categories, as follows: 100, 200, 300, 400, and 500 RMB. These were the basic categories of the premium that were designed by the NRPS. However, local governments could introduce more categories to the program, based on their regional economic status. The higher the premium the individuals paid, the more pension payment they would get when they were age-eligible for the pension benefits. With different premium levels that each person selected, the government provided different levels of subsidy, and the minimum amount of the total subsidy that was provided by the central and local governments should not be less than 30 RMB per person, per year, while the maximum amount should have been no more than 50 RMB. For different regions, the percentage of the premium subsidy that was provided by the central government was different. Generally, this percentage could be classified into four categories, as follows: 20%, 40%, 60%, and 80%, and the amount that was subsidized from the central government was determined by the local government, based on the regional economic status. For example, if an individual selected the 100 RMB premium, he/she would get the minimum amount of a premium subsidy, 30 RMB. Given that the percentage of that subsidy that was covered by the central government was 80%, then the subsidy from the central government was 24 RMB (30 * 80% = 24 RMB) and the remaining 6 RMB would be paid by local government. Finally, the payment method of the pension benefits was different from the pilot rural pension program. The pension that was paid out from the NRPS consisted of two parts, as follows: benefits from the personal contribution and a basic pension benefit. Benefits from personal contributions were related to the premium that was paid by individuals, and the higher the premium that the individuals paid, the more benefit they would get. The basic pension benefit started at 55 RMB per month initially, and increased to 70 RMB in 2014, in accordance with the GDP growth and inflation. The amount of the basic pension varied considerably across counties; it was as high as about 370–380 RMB per month in Beijing and Shanghai in 2012, but it was only 55 RMB per month in less developed areas [2]. For the benefit from the personal contribution, the more premium the participants paid, the more benefits they would receive, and all of the pensioners also got an additional basic payment, regardless of what premium level they selected to pay. This benefit payment method represented the first time that the Chinese government had undertaken major financial support for a rural pension system [20].

### 2.2. Data and Variables

The data that were used in this paper were obtained from the CHARLS. The CHARLS was a nationwide survey aiming to collect personal and familial information regarding the elderly population. People of the age 45 and older were randomly selected to be interviewed in the survey. The baseline national wave was fielded in 2011, and the follow up waves were conducted every other year. The sampling process of the CHARLS included three stages, as follows: county-level sampling, neighborhood-level sampling, and household-level sampling. In the stages of the county-level and neighborhood-level sampling, the multi-stage stratified PPS (probabilities proportional to size) sampling was adopted to select 150 counties/districts and 450 villages/communities from 28 provinces (the Tibet Autonomous Region was excluded). After selecting the neighborhoods, a software package (CHARLS-GIS), which was an innovation of CHARLS, was created to randomly sample 80 households from each neighborhood. In the 2013 wave, 17.6% of the 2011 wave respondents exited the survey, while 23.7% of the 2013 wave respondents exited the survey in 2015. Since only the 2015 wave survey was applied in our study, the estimation results would not be affected by the exit of the respondents between those two waves. 

The questionnaire of the CHARLS contained six parts, as follows: demographic background; family information; health status and functioning; health care and insurance; work, retirement, and pension; and income, expenditures, and assets, which provided rich information regarding economic standing, physical and psychological health, demographics, and social networks of aged persons. Data about the outcome variable-consumption could be drawn from the questionnaire of income, expenditures, and assets. Besides the information on consumption, individual demographic background, health status, and some household characteristics were also included in our analysis. Information on socio-demographic characteristics of the elderly could be identified from the demographic background questionnaire, which included age, gender, and educational attainment. With respect to the information about NRPS participation and pension payment, we relied on the questionnaire of work, retirement, and pension. Chronic diseases of the respondents were reported in the part on health status and functioning, while saving and household income could be obtained from the questionnaire on income, expenditures, and assets.

In 2009, the NRPS was only first introduced to 320 pilot counties (10% of the total counties), and it was not till 2013 that the NRPS covered all of the counties. To avoid policy selection bias, this study used the latest 2015 waves of the CHARLS data, the year in which the NRPS covered the whole country, to update studies of evaluating the NRPS performance. Since the NRPS was targeted to the elderly population in rural areas, this study used a restricted sample that comprised the rural elderly, and excluded observations regarding pension programs outside of the NRPS. Our main study sample contained 9883 elderly individuals. The summary statistics of all of the variables are represented in Table 1, showing that 43.7% of the respondents received a pension income, and only 33.2% of the respondents reported they had personal savings. On average, the food expenditure of the rural elderly accounted for about 46.5% of the total expenditure. Non-food expenditure consisted of expenditure on clothing and bedding, travelling, heating, consumption of durable goods, education and training, medical treatment, fitness, beauty, automobiles, property management fees, and donation. This indicated that an increased income would largely improve their food consumption ability. Since the CHARLS data did not provide information on how long the respondent had been diagnosed with the chronic diseases, the variable of ‘chronic disease’ was coded as a dummy indicator, which equaled 1 if the respondent reported that she/he was diagnosed with at least one of the following chronic diseases: hypertension, dyslipidemia, diabetes or high blood sugar, coronary heart disease, heart attack, stroke, kidney disease, arthritis or rheumatism, and asthma. Therefore, one of the limitations of our study was that the estimation result could not differentiate the effect of the NRPS on the elderly with moderate chronic diseases from those with severe chronic diseases. Table 1 shows that 70% of the respondents reported that they were diagnosed with at least one of the listed chronic diseases. Other individual characteristics included age, gender, educational attainment, and labor supply. For educational attainment, about 65% of the respondents had no formal education or had only attended elementary school. Although a large proportion of the respondents had low or little educational attainment, interviewers would provide assistance if they had difficulties in understanding the questionnaire during the face-to-face interview. The data of food prices that were used in this paper were collected from each province’s Bureau of Commodity Price and Development and Reform Commission. All of the prices were adjusted to the 2015 RMB.

### 2.3. Methodology

#### 2.3.1. Food Poverty Line and Vulnerability to Food Poverty Threshold

Before proceeding to the empirical strategy, we need define some concepts first. In the analysis of food poverty, a cut-off point, known as the poverty line, needed to be determined. The construction of a food poverty line was relatively subjective, and, to a large extent, depended on data availability. From existing studies, four approaches were applied in the setting of the food poverty line, as follows: direct calorie intake (DCI) method, food energy intake (FEI) approach, cost of basic needs (CBN) approach, and arbitrary choice of index (ACI) method [21]. Among all of these methods, the FEI approach was supposed to be the most appropriate in food poverty analysis [11]. To apply this approach, information on prices and energy conversion for different food items, or alternative information on food expenditure and calorie consumption needed to be known. However, in the absence of information on calorie consumption, which was the case of this study, with the spirit of the FEI method, the least cost approach was one of the most popular and robust approaches [11]. The basic idea of this approach was the selection of food items from the commonly consumed food list that satisfied the calories requirement of the per adult equivalence per day with minimum cost. Following Soden and Fletcher (1992) [22], the nutritional constraints in terms of calories and the suggested healthy diet pattern could be easily incorporated into a linear programming technique to determine the food poverty line. The average 2300 kilocalories per adult per day, provided by China’s National Health and Family Planning Commission, was adopted in this study as the nutritional constraint, and the healthy diet pattern was based on the Dietary Guidelines for Chinese Residents (2016) [23]. According to the 2016 Dietary Guidelines, Chinese residents were recommended to intake 25–30 g cooking oil, no less than 300 g dairy product, no less than 25 g soyfoods, 40–75 g meat, 40–75 g fish or shrimp, 40–50 g eggs, 300–500 g vegetables, 200–350 g fruits, and 250–400 g cereals.

Our linear programming model that was used to determine the poverty line was presented as follows:

(1) $$Minimise:\;\sum_{i=1}^{k}p_{i}F_{i}$$

(2) $$Subject\;to:\{\begin{array}{c} \sum_{i=1}^{k}e_{i}F_{i}\geq R\\ \sum_{i=1}^{k}c_{i1}F_{i}\geq h_{1}\\ \begin{array}{c} \vdots \\ \sum_{i=1}^{k}c_{ij}F_{i}\geq h_{j}\\ F_{i}\geq 0 \end{array} \end{array}$$

where $F_{i}$ is a vector of food items; $p_{i}$ is a vector of the unit prices of the food items; k represents the number of commonly consumed food items; $e_{i}$ is a vector of the calories per unit of the food (the data about food calories were obtained from The Composition of Chinese Foods [24]) $F_{i}$; R denotes the calories requirement for an adult equivalence per day; $c_{ij}$ is the quantity of nutrient j per unit of food $F_{i}$; $h_{j}$ is the recommended amount of nutrient j, which ensures the selected food items satisfy the requirements of the healthy diet pattern proposed by the Dietary Guidelines for Chinese Residents (2016). As indicated in other literature, food wastage accounted for about 10% of the total energy [11,25], we multiply the calories constraint in the linear programming model by a factor of 1.1. Anyone with food consumption lower than the least cost expenditure that was obtained from the linear programming was considered poor.

Vulnerability to food poverty was defined as the probability that an individual’s food consumption would fall below some benchmark, here it was the food poverty line [21,26]. Following previous studies that accepted a vulnerability threshold of 0.5 [21,27] for the advantage of improved prediction, we also defined the food poverty vulnerability threshold as 0.5.

#### 2.3.2. Measuring Food Poverty

Defining an individual with food consumption lower than the least cost expenditure that was obtained from the linear programming as poor, the next action was the measurement of food poverty. Although many poverty measures had been adopted in the literature, the Foster–Greer–Thorbecke (FGT) family of poverty indices was the most popular poverty measure, which provided a distribution sensitive measure through the choice of a ‘poverty aversion’ parameter [11,28]. The FGT family of poverty indices was given as follows:

(3) $$P_{\alpha }=\frac{1}{N}\sum_{i=1}^{np}{\left[\frac{Z-E_{i}}{Z}\right]}^{\alpha }$$

$P_{\alpha }$ is the food poverty index of interest; N is the number of individuals, and np is the number of individuals below the food poverty line; Z denotes the food poverty line; $E_{i}$ is the daily per adult equivalent food expenditure; and α, with a value of 0, 1, or 3, is the poverty aversion parameter. When α is set at 0, the food poverty index reduces to the poverty headcount index, measuring the proportion of individuals that have been identified as poor; when α is set at 1, the food poverty index reduces to the poverty gap index, which measures how far those who have been identified as poor fall below the food poverty line; when α is set at 2, the food poverty index reduces to the squared poverty gap index, which measures the extent of inequality among the poor. This study presented the results of food poverty incidence by setting α at 0.

#### 2.3.3. Pension Income and Food Expenditure

As people aged, their work activities and income gradually declined, and most of their expenditure was financed by personal saving and transfers from adult children. With the higher poverty rate, the rural elderly often had to curb their spending on food in order to have money for other expenses, and so they experienced inadequate food intake [29]. The NRPS pension payment, which directly raised the income, may have changed the elder’s consumption pattern. To explore the causal relationship between pension income and consumption, we employed a regression discontinuity (RD) design, which accounted for the endogeneity of pension receipt by taking advantage of a discontinuity in NRPS pension receipt that was induced by age eligibility.

During implementation, with different preferences of local government to payment time (some local government paid the pension monthly, but many local governments preferred to pay quarterly or yearly), many elderly people would not receive a pension income immediately after their 60th birthday. Therefore, a fuzzy RD design was adopted in our analysis. The estimation of the fuzzy RD design was often carried out by a two-stage least squares (2SLS) method, using $I\left(age_{i}\geq 60\right)$ as the instrument for pension. The parametric equations of the fuzzy RD design are defined as follows:

(4) $$lnE_{i}=\alpha_{1}+\tau_{1}pension_{i}+\omega_{1}C_{i}+\epsilon_{i}$$

(5) $${pension}_{i}=\alpha_{0}+\tau_{0}{}_{\;}I\left({age}_{i}\geq 60\right)+f_{0}\left({age}_{i}-60\right)+\omega_{0}C_{i}+\varepsilon_{i}$$

$lnE_{i}$ is the log of daily food expenditure of individual i; $pension_{i}$ a dummy of pension receipt; $f\left(age_{i}-60\right)$ is a polynomial function of the normalized age; $I\left(age_{i}\geq 60\right)$, the instrument for pension receipt, is an indicator of age above the 60 cutoff; and $C_{i}$ represents covariates. The daily food expenditure of individual i is obtained by dividing the seven-day food expenditure per household by 7 and then by the number of adults at table in the household. The number of adults at table is measured by the adult equivalents in the households, which is calculated based on the World Health Organization adult equivalence scale (presented in Table A1). This scale is derived from detailed studies of the nutritional requirements of males and females of different ages in developing countries [30,31]. The fitted values of the pension receipts that were obtained from the first stage were then applied in the feasible generalized least squares (FGLS) method, which was adopted to analyze the vulnerability to food poverty.

#### 2.3.4. Vulnerability to Food Poverty

Poverty is a dynamic phenomenon, which may be influenced by exogenous shocks, like changes in income sources. The econometric methods for analyzing vulnerability to food poverty involved three approaches, namely: vulnerability as expected poverty (VEP), vulnerability as low expected utility (VEU), and vulnerability as uninsured exposure to risk (VER) [21,32]. In our study, vulnerability to food poverty, a different concept from food poverty, measured the probability that an individual, whether currently poor or not, may be poor in the near future. For this purpose, the VEP approach was selected. According to VEP, the probability that individual i would be food poor at time t+Δ was as follows:

(6) $$V_{i,t}=Pr(lnE_{i,t+\Delta }<lnZ)$$

$V_{i,t}$ is the vulnerability to food poverty of individual i at time t; $E_{i,t+\Delta }$ is the daily food expenditure of individual i at time t+Δ*;* and Z denotes the food poverty line. Individual $i^{\prime }s$ food expenditure is determined by a number of individual and household characteristics, and is given by the following:

(7) $$lnE_{i}=\alpha X_{i}+\mu_{i}$$

$X_{i}$ is a vector of pension receipt and other individual and households characteristics, and $\mu_{i}$ is the idiosyncratic error term with mean zero and normal distribution. To account for the heteroscedasticity of the cross-sectional data, a three-stage FGLS procedure that was proposed by Amemiya (1977) [33] was adopted. The steps of the FGLS procedure are described in Appendix B. Replacing the dependent variable of the fuzzy RD design, described in Equation (5), with food poverty and vulnerability, the effects of pension income on food poverty and vulnerability were investigated.

## 3. Results

### 3.1. Food Poverty and Vulnerability Decomposition

In view of the food price variation across provinces, a province-specific food poverty line was constructed using the least cost approach. Thresholds of energy intake varied in different studies. The World Health Organization considered 2850 kilocalories as the required daily intake. Masood et al. (2016) [34] applied 3000 kilocalories and Zereyesus et al. (2017) [27] used 2900 kilocalories as the minimum daily energy intake for a moderately active adult equivalent. This study applies a subjective 2530 kilocalories as the threshold of energy intake, which is the average energy consumption of Chinese rural residents multiplying a factor of 1.1 to account for food wastage. Adding the Dietary Guidelines for Chinese Residents (2016) [23] proposed healthy diet requirements to the linear programming, the least cost food expenditure was estimated. An individual was defined as poor in food when his/her daily food expenditure was lower than the province specific food poverty line. Vulnerability to food poverty was defined as the probability that an individual would, if currently non-food poor, fall in food poverty in the future, or if currently food poor, remained in food poverty. Vulnerability to food poverty, unlike the concept of food poverty, was a more future-oriented concept that took the changes in an individual’s future welfare into account [21]. This study defined an individual as vulnerable to food poverty if he/she had a probability higher than 50% of falling below the food poverty line.

**Figure 1:**
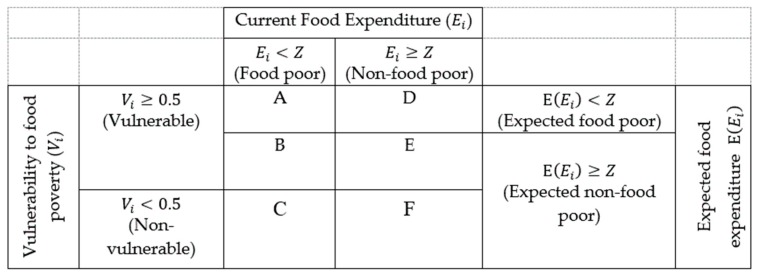
Categorization of food poverty and vulnerability to food poverty. Note: $E_{i}$ is the current food expenditure, $E\left(E_{i}\right)$ is the expected food expenditure, $V_{i}$ represents the vulnerability to food poverty, and Z is the least cost food poverty line.

Following Maeda and Tscherning [35], Ward [36], and Azeem et al. [37], Figure 1 shows the categorization of food poverty and vulnerability, which helped to investigate the various manifestations of current poverty, future expected poverty, and current vulnerability status. Six overlapping categories (*A* to *F*) of individuals were grouped in Figure 1, based on their current food expenditure ($E_{i}$), expected food expenditure $\left(E\left(E_{i}\right)\right)$, and vulnerability to food poverty $\left(V_{i}\right)$. In this figure, the currently food poor individuals (those individuals whose current food expenditure lay below the food poverty line) were represented by area *A* + *B* + *C*, while the non-food poor individuals were denoted by area *D* + *E* + *F*. The current food poor individuals were further decomposed into those that were chronically or structurally food poor, as represented by area *A*, and those that were transient food poor, as represented by area *B* + *C*. The chronically food poor individuals were those who were currently food poor, and their structural characteristics suggested that they were also expected to be poor, while the transient food poor individuals were those who were currently food poor, but not expected to be poor. The non-food poor individuals were classified as high vulnerable non-food poor (HVNP) and low vulnerable non-food poor (LVNP), which are show in Figure 1 as areas *D* + *E* and F, respectively. Vulnerable individuals—individuals whose probability of falling below the food poverty line was greater than our vulnerability threshold of 0.5—were denoted by area *A* + *B* + *D* + *E*. These individuals could be classified as those who were vulnerable because of low expected food expenditure (LEE, area *A* + *D*) and those who were vulnerable because of highly variable food expenditure (HVE, area *B + E*).

**Figure 2:**
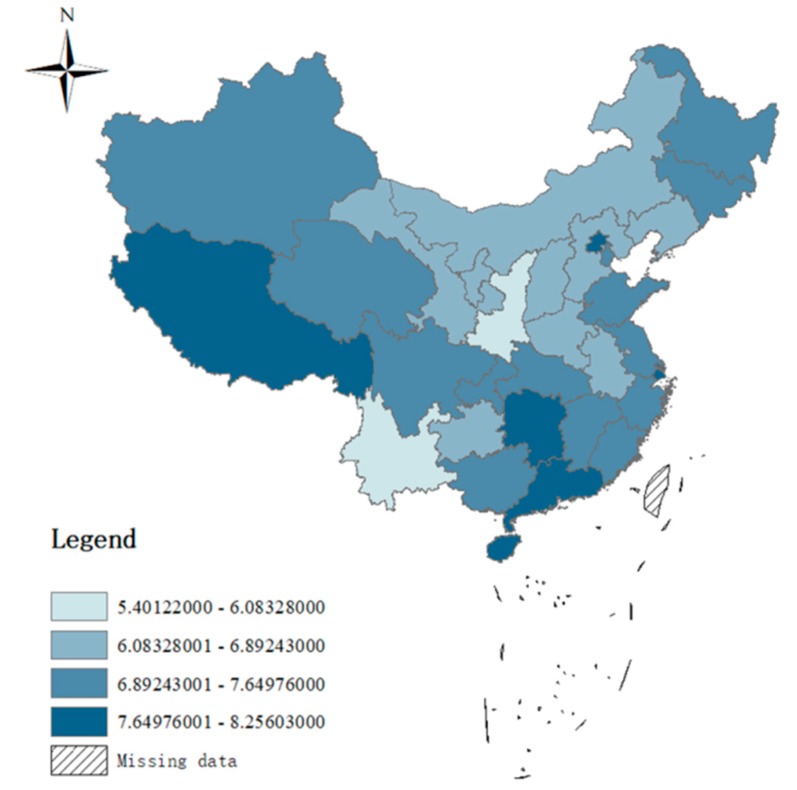
Distribution of food poverty lines.

Applying a 2530 kilocalorie constraint in the linear programming, the food poverty line for each province was calculated. The distribution of the food poverty line across the whole country is shown in Figure 2, with the lowest food poverty line of 5.4 yuan/day in Shaanxi Province and the highest poverty line of 8.25 yuan/day in Shanghai. The average food poverty line of the whole country was 7.2 yuan/day. Figure 2 indicates that the central area had lower food poverty lines than the other parts of the country.

According to the estimated province-specific poverty lines, each observation in our sample could be assigned to one of the six categories that are described in Figure 1. The headcount index was applied to measure the disparities of the food poverty incidence between the elderly with chronic diseases and those without any chronic diseases. Table 2 shows that the overall food poverty incidence in rural China was 39.9%, of which 10.3% were chronic food poor, while 29.6% were transient food poor. For the elderly with chronic diseases, the food poverty incidence was 41.9%, with 11% being chronic food poor and 30.9% being transient food poor. This indicated that 11% of the elderly with chronic diseases were currently food poor and their structural characteristics suggested that they were also expected to be poor, while 30.9% of them were currently food poor, but were not expected to be poor in the future. Compared with the elderly without any chronic diseases, Table 2 showed that the elderly with chronic diseases had a higher food poverty incidence. This may have been explained by the fact that poor physical health and extra expense on disease treatment limited the resources that were available for food acquisition by the elderly with chronic diseases. If we subscribed to the notion that transient poverty was primarily because of unanticipated shocks, the higher incidence of transient poverty among the elderly with chronic diseases suggested that they were more likely to be affected by unanticipated shocks. The results of the food poverty classification could also provide policy implications, since the chronically poor elderly needed targeted interventions in health, infrastructure, land reforms, and old age benefits [37], while interventions related to risk management should have targeted the transient poverty population [31,38].

The estimated vulnerability to food poverty in rural China was 33.5%, and 22.2% were vulnerable because of the low expected food expenditure, and 11.3% were vulnerable because of the high variance in the expected food expenditure. Regarding to the two subsamples, the elderly with chronic diseases were more vulnerable to food poverty than the elderly with good heath, which indicated that the current health status played a crucial role in determining the household’s vulnerability to poverty. Food consumption losses were found to be associated with health related shocks [21,37]. As general health status declined, the elderly with chronic diseases were exposed to a higher risk of losing jobs because of their suffering symptoms, and therefore faced unstable income sources. With limited financial resources, the elderly with chronic diseases have lower ability of bearing risks, and hence were less resilient to unanticipated shocks in the future (e.g., health shocks) [35]. Health shocks were unpredictable and posed a great challenge that any household/individual had to face [21]. Somi et al. (2009) [39] indicated that health shocks and their associated costs could force individuals to substitute consumption expenditure for health care. With relative poor health status, the elderly with chronic diseases were more likely to experience health shocks, leading them to be more vulnerable to food poverty. According to the categorization in Figure 1, we were able to classify the non-food poor into two groups, HVNP and LVNP. For the HVLP elderly, although their income did not fall below the food poverty line currently, they were expected to be poor in the future. Table 3 shows that the HVNP elderly with chronic diseases were 2.1% higher than those without any chronic diseases, which indicated that an advantage in health status could stabilize income sources and set grounds for a resilient future. This was consistent with the result in Table 1 that the elderly with chronic diseases were more likely to be affected by unanticipated shocks.

### 3.2. Pension Income, Food Consumption, and Vulnerability to Food Poverty

To take advantage of the age discontinuity of the pension payment, a fuzzy RD design, estimated using a dummy for ‘age above 60’ as the instrument for pension income, was applied to identify the effects of pension income on food poverty and vulnerability. Since high-order polynomials of age function could be misleading in a parametric RD estimation [40], we use a quadratic polynomial age function. To discover the effects of NRPS on different population groups and to evaluate the policy performance, besides the estimates applying all of the observations, we also duplicated the estimation applying to two subsamples, (1) the elderly with chronic diseases and (2) the elderly without any chronic diseases. When applying a parametric RD estimation, the choice of the bandwidth was critical. Our main results were focused on the rural elderly aged between 50 and 70. To check the robustness of the results, sensitivity tests were carried out using multiple bandwidth specifications. The RD estimates of the effect of the NRPS on food poverty and vulnerability applying different samples are shown in Table 4. Overall, the empirical results showed that pension income was significantly and negatively associated with food poverty. The pension income reduced the probability that the elderly would fall below the food poverty line by 11.7%. This result aligned with previous research showing a positive relationship between pension income and food consumption. Applying the data from China Family Panel Studies, Wang and Zhou [41] showed that pension benefit increased expenditure on non-durable goods by 15.9%, mainly because of the increase in food consumption. Zhao et al. [42] used the CHARLS 2011 and the 2013 survey data and showed that, compared with those that were not covered by any public pension program, the individuals enrolled in the public pension system tended to consume more food. Regarding the vulnerability to food poverty, Table 4 shows that pension income significantly decreased vulnerability by 17.7%, indicating that the elderly with pension income were less vulnerable to food poverty in the future. As an additional source of income, the NRPS pension income improved the elderly’s food consumption with increased adaptive capacity to consumption variation.

Since income was a predictor of an individual’s or a household’s ability to access enough nutritious foods, pension income was expected to have greater impacts on the elderly with chronic diseases. The estimation results in Table 4 identified that the NRPS played a more important role for the elderly with chronic diseases—pension income decreased the food poverty and vulnerability among the elderly with chronic diseases by 12.9% and 16.8% respectively, while it had no significant effect on the elderly without any chronic diseases. This may have been explained by the fact that, compared with the well-documented generous pension program in South Africa that pays twice the average income, the NRPS in China provided a modest payment to the pensioners. With a higher income, the consumption of the elderly in good health could not be significantly affected by this modest payment. Since food poverty incidence and vulnerability was unequally distributed between the elderly with chronic diseases and those without any chronic diseases, the health status needed to be considered when improving the NRPS’s role in poverty reduction.

Several control variables were also included in the model. Applying a mean age of 59.34, Table 4 shows that age had a significantly positive correlation with food poverty and vulnerability, indicating that food poverty incidence and vulnerability increased significantly with aging. Table 4 also indicates that females were less vulnerable to food poverty than males, which was consistent with the finding that female-headed households were relatively more food-secure compared with male-headed households [37]. The elderly with a higher educational attainment were less likely to be food poor. Individuals with a higher educational attainment were more qualified for higher income jobs, and they were more able to deal with expenditure uncertainty in the future. Current working status and saving correlated with food poverty and vulnerability negatively. The expected food expenditure increased significantly with household income, as a result of the fact that in China, especial in rural China, transfer from family members was the main financial support for old people. With a higher total household income, the elderly were less vulnerable to future food poverty. Region fixed effects indicated that the elderly in more developed regions had lower food poverty incidence and were less likely to fall below the food poverty line in the future.

## 4. Robustness Checks

**Figure 3:**
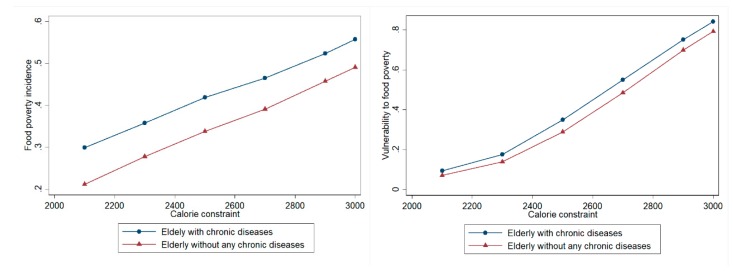
Food poverty incidence and vulnerability to food poverty for various calorie constrains.

To have more confidence in the estimation results, a series of robust checks were incorporated. The first concern was given to the subjective 2530 kilocalories constraint in the estimation of food poverty lines. We created six adult equivalence per day calorie constraints, ranging from 2000 kilocalories to 3000 kilocalories, which gave us six least cost food expenditures. Applying these least-cost expenditures as food poverty lines, Figure 3 gives the food poverty incidence and vulnerability to food poverty for both the elderly with chronic diseases and those without any chronic diseases. The results showed that the distribution of vulnerability and food poverty between different groups was robust: the elderly with chronic diseases experienced higher food poverty incidence and were more vulnerable to food poverty for all adult equivalence per day calorie constraints than the elderly without any chronic diseases. However, the magnitude of food poverty incidence and vulnerability estimates were sensitive to the choice of calorie constraint, and with a higher calorie constraint, the magnitude of food poverty incidence and vulnerability estimates were larger for both population groups.

The second concern was the validity of the RD design in estimating the effect of pension income on food poverty and vulnerability. To make the RD design valid, the age distribution should not have been determined in the presence of knowledge about the corresponding cut-point; otherwise, it could be manipulated to include or exclude specific candidates, which would bias the estimation results. In the present case of NRPS, this seemed unlikely as birthday information on the registration cards were recorded far ahead of the pension reform [1]. Although, given that a window of bandwidth around the cut-point a fuzzy RD model could be estimated straightforwardly, it was challenging to choose this bandwidth. The tradeoff between bias and precision was a major motivator of bandwidth selection—larger bandwidths reduced the noisiness of estimates at the expense of introducing bias from data points far from the cutoff for treatment [43]. To test the robustness of our RD estimation results, besides the sample of the elderly that was aged within 10 years around the 60 cutoff, we also duplicated the estimation procedures by applying samples of elderly aged 52–68 and 48–72. The estimates using different bandwidth specifications were consistent, confirming our results were robust to the bandwidth selection (Table A2 and Table A3).

## 5. Conclusions

The NRPS, which was launched in 2009 in rural China, aims to ensure the well-being of the rural elderly by providing a pension payment to the rural elderly at the age of 60. In rural China, the elderly with chronic diseases experience a higher poverty incidence and are more vulnerable to food poverty than those without any chronic diseases. Therefore, to make the NRPS more efficient in improving the well-being of the rural elderly, it is important to evaluate the performance of the NRPS in consumption promotion and food poverty reduction among different population groups. Applying a nationally representative micro survey dataset, this study estimates the effect of pension income on food poverty and vulnerability among the elderly.

The concept of vulnerability as a characteristic of well-being is an important indicator for policy-makers to effectively target populations at risk, with assistance so as to prevent welfare losses in the future [36]. Using a three-stage FGLS procedure, the vulnerability to food poverty, defined as the probability of an individual falling below the food poverty line in the future, is estimated. Consistent with previous studies demonstrating that better health status is an important vulnerability-reducing variable [21], our results show that food poverty incidence and vulnerability of the elderly with chronic diseases in rural China is 41.9% and 35% respectively, which is 8% and 6% higher, respectively, than the elderly in good health. To investigate the effect of the NRPS on food poverty and vulnerability, a fuzzy RD design is employed using the age discontinuity of the pension payment. Overall, the empirical results show that pension income is significantly and negatively associated with food poverty and vulnerability, indicating that the elderly with a pension income are less vulnerable to food poverty in the future. Regarding the different population groups, we have found that pension income decreases the probability of being food poor and the vulnerability to food poverty among the elderly with chronic diseases by 12.9% and 16.8% respectively, while it has no significant effect on the elderly without any chronic diseases. The major policy implication of our findings is that it is important to realize that the food poverty incidence and vulnerability is different among different population groups. These disparities should be taken into account when designing polices of poverty reduction.

## Figures and Tables

**Table 1:** Descriptive Statistics. NRPS—New Rural Pension Scheme.

Variable	Definition	Mean	Standard Deviation
Food Expenditure	Log of food expenditure	2.141	1.044
Total Expenditure	Log of total expenditure	2.456	1.501
NRPS Pension	=1 if received pension income	0.437	0.496
Age	Age in year	57.328	10.113
Male	=1 if gender is male	0.470	0.499
Illiterate	=1 if has no formal education	0.258	0.437
Elementary School	=1 if attended elementary school or under	0.391	0.488
Middle School	=1 if attended middle school or under	0.170	0.375
High School	=1 if attended high school or higher	0.051	0.221
Working	=1 if is currently working	0.754	0.431
Saving	=1 if have personal saving	0.332	0.471
Chronic Disease	=1 if diagnosed with chronic disease	0.704	0.456
Household Income	Annual household income (10,000 yuan)	1.443	10.609
N	Number of Observations	9883	

Source: China Health and Retirement Longitudinal Survey 2015.

**Table 2:** Food poverty incidence in rural China.

	Food Poverty Incidence	Chronic Food Poverty	Transient Food Poverty
Overall	0.399	0.103	0.296
Elderly with Chronic Diseases	0.419	0.110	0.309
Elderly without Chronic Diseases	0.338	0.082	0.256

**Table 3:** Vulnerability Decomposition.

	Magnitude			Source	
	VFP	HVNP	LVNP	LEE	HVE
National	0.335	0.118	0.482	0.222	0.113
Elderly with Chronic Diseases	0.350	0.124	0.457	0.234	0.116
Elderly without Chronic Diseases	0.290	0.103	0.559	0.185	0.105

Note: Table 3 describes vulnerability decomposition results. VFP—vulnerability to food poverty; HVNP—high vulnerable non-food poor; LVNP—low vulnerable to non-food poor; LEE—low expected food expenditure; HVE—high variability of food expenditure.

**Table 4:** Effects of pension income on food poverty and vulnerability.

	Overall		The Elderly with Chronic Disease		The Elderly without Any Chronic Disease	
	(1)		(2)		(3)	
	Food Poverty	vulnerability	Food Poverty	Vulnerability	Food Poverty	Vulnerability
Pension Income	−0.117 *	−0.177 ***	−0.129 *	−0.168 ***	−0.121	−0.185
Age − 60	0.011 **	0.014 ***	0.012 **	0.011 ***	0.009 *	0.023 ***
(Age − 60)^2^	−0.001 **	−0.001	−0.001 ***	−0.002	−0.001	−0.002
Male	−0.001	0.019 ***	−0.002	0.013*	−0.001	0.020 *
Elementary School	−0.019	−0.003	−0.025	−0.005	−0.005	−0.012
Middle School	−0.004	−0.020 **	−0.002	−0.027 **	−0.006	−0.009
High School	−0.035 *	−0.054 ***	−0.037 **	−0.050 ***	−0.023 **	−0.056 **
Working	−0.034 *	−0.209 ***	−0.031 ***	−0.208 ***	−0.048 *	−0.251 ***
Saving	−0.024 *	−0.026 ***	0.024 ***	−0.018 **	−0.017 **	−0.054 ***
Household Income	−0.002 ***	−0.001 **	−0.002 ***	−0.001 **	−0.012 ***	−0.003 *
East Region	−0.105 ***	−0.169 **	−0.108 **	−0.162 **	−0.088 **	−0.164 **
Central Region	−0.050 **	−0.141 **	−0.048 ***	−0.133 **	−0.056 *	−0.139 *
No. of obs.	6211		4670		1541	
$R^{2}$	0.487	0.237	0.487	0.224	0.488	0.231

Note: Table 4. Describes the effects of pension income on food poverty and vulnerability applying RD regression for different subsamples. In the parametric fuzzy RD regression, a second polynomial is used. Model (1) shows the estimation results using all of the elderly aged between 50 and 70; model (2) shows the estimation results using the elderly with chronic diseases; and model (3) shows the estimation results using the elderly without any chronic diseases. * significant at 10% level; ** significant at 5% level; *** significant at 1% level.

## Abbreviations

NRPS	New Rural Pension Scheme
FGLS	feasible generalized least squares
CHARLS	China Health and Retirement Longitudinal Survey
RD	regression discontinuity
DCI	direct calorie intake
FEI	food energy intake
CBN	cost of basic needs
ACI	arbitrary choice of index
FGT	Foster–Greer–Thorbecke
2SLS	two-stage least squares
VEP	vulnerability as expected poverty
VEU	vulnerability as low expected utility
VER	vulnerability as uninsured exposure to risk
OLS	ordinary least square
HVNP	high vulnerable non-food poor
LVNP	low vulnerable non-food poor
LEE	low expected food expenditure
HVE	highly variable food expenditure
PPS	probabilities proportional to size

## Appendix A

**Table A1:** Adult equivalence scale.

Age	Male Weight	Female Weight
0	0.33	0.33
1	0.46	0.46
2	0.54	0.54
3–4	0.62	0.62
5–6	0.74	0.70
7–9	0.84	0.72
10–11	0.88	0.78
12–13	0.96	0.84
14–15	1.06	0.86
16–17	1.14	0.86
18–29	1.04	0.80
30–59	1.00	0.82
60+	0.84	0.74

Note: The equivalence scale is based on a World Health Organization equivalence scale quoted by Dercon (1998) [44].

**Table A2:** Effects of pension income on food poverty and vulnerability of the elderly aged between 48 and 72.

	Overall		The Elderly with Chronic Diseases		The Elderly without Any Chronic Diseases	
	(1)		(2)		(3)	
	Food Poverty	Vulnerability	Food Poverty	Vulnerability	Food Poverty	Vulnerability
Pension Income	−0.101 *	−0.159 ***	−0.099 *	−0.183 ***	−0.128	−0.064
Age − 60	0.009 **	0.016 ***	0.010 ***	0.015 ***	0.010 *	0.017 ***
(Age − 60)^2^	−0.001 *	−0.001	−0.001 *	−0.001	−0.001	−0.001
Male	−0.006	0.019 ***	−0.005	0.022 **	−0.004	0.025 *
Elementary School	−0.009	−0.051	−0.019	−0.051	−0.020	−0.044
Middle School	−0.003	−0.037 *	−0.005	−0.041 **	−0.002	−0.022
High School	−0.019 **	−0.056 ***	−0.021 **	−0.059 ***	−0.012 ***	−0.049 **
Working	−0.027 *	−0.156 ***	−0.022 *	−0.159 ***	−0.044 *	−0.173 ***
Saving	−0.024 *	−0.077 ***	−0.019 **	−0.079 ***	−0.034 **	−0.071 ***
Household Income	−0.002 ***	−0.001 ***	−0.002 ***	−0.001 ***	−0.008 ***	−0.001 *
East Region	−0.114 **	−0.196 *	−0.115 **	−0.199 **	−0.102 **	−0.171 *
Central Region	−0.062 ***	−0.143 **	0.055 **	−0.143 **	−0.076 **	−0.132 **
No. of obs.	7304		5446		1858	
$R^{2}$	0.486	0.216	0.486	0.242	0.486	0.232

Note: Table A2 describes effects of pension income on food poverty and vulnerability applying RD regression for different subsamples. In the parametric fuzzy RD regression, a second polynomial is used. Model (1) shows the estimation results using all of the elderly aged between 48 and 72; model (2) shows the estimation results using the elderly with chronic diseases; and model (3) shows the estimation results using the elderly without any chronic diseases. * significant at 10% level; ** significant at 5% level; *** significant at 1% level.

**Table A3:** Effects of pension income on food poverty and vulnerability of the elderly aged between 52 and 68.

	Overall		The Elderly with Chronic Diseases		The Elderly without Any Chronic Diseases	
	(1)		(2)		(3)	
	Food Poverty	Vulnerability	Food Poverty	Vulnerability	Food Poverty	Vulnerability
Pension Income	−0.156 *	−0.117 **	−0.187 *	−0.128 **	−0.190	−0.153
Age − 60	0.015 *	0.009 **	0.017 *	0.008 *	0.016 *	0.020 *
(Age − 60)^2^	−0.002 **	−0.001	−0.002 **	−0.001	−0.001	−0.001
Male	−0.010	0.014 *	−0.015	0.010 *	−0.009	0.032 *
Elementary School	−0.017	−0.034	−0.015	−0.035	−0.005	−0.044
Middle School	−0.005	−0.038 **	−0.001	−0.043 **	−0.016	−0.029
High School	−0.052 **	−0.066 ***	−0.053 *	−0.062 ***	−0.043 **	−0.080 **
Working	−0.036 **	−0.180 ***	−0.035 **	−0.157 ***	−0.044 *	−0.245 ***
Saving	−0.018 *	−0.062 ***	−0.016 *	−0.052 ***	−0.014 **	−0.075 ***
Household Income	−0.003 ***	−0.001 ***	−0.002 ***	−0.001 ***	−0.004 ***	−0.002 *
East Region	−0.108 **	−0.158 ***	−0.116 **	−0.139 **	−0.075 ***	−0.155 *
Central Region	−0.054 ***	−0.139 *	−0.054 **	−0.124 **	−0.045	−0.140 *
No. of obs.	5071		3852		1219	
$R^{2}$	0.490	0.220	0.489	0.211	0.490	0.222

Note: Table A3 describes effects of pension income on food poverty and vulnerability applying RD regression for different subsamples. In the parametric fuzzy RD regression, a 2nd polynomial is used. Model (1) shows the estimation results using all of the elderly aged between 52 and 68; model (2) shows the estimation results using the elderly with chronic diseases; and model (3) shows the estimation results using the elderly without any chronic diseases. * significant at 10% level; ** significant at 5% level; *** significant at 1% level.

## Appendix B

Steps of the three-stage FGLS:

Using estimates from Equation (7), the venerability to food poverty of individual i is estimated as follows:

(A1) $${\hat{V}}_{i,t}=Pr\left(lnE_{i,t+\Delta }<lnZ|X_{i,t}\right)=\Phi \left(lnZ-\hat{\alpha }\hat{\sigma }X_{i}\right)$$

${\hat{V}}_{i,t}$ is the estimated vulnerability to poverty; Φ(·) is the cumulative density of the standard normal distribution, and $\hat{\sigma }$ is the estimated standard error from Equation (7).

To account for the heteroscedasticity of the cross-sectional data, a simple functional form that relates the variance of the expenditure function to individual and household characteristics is shown in Equation (A2).

(A2) $$\sigma_{\mu ,i}^{2}=\beta X_{i}+\theta_{i}$$

The steps of the FGLS procedure are described as follows:

Firstly, Equation (7) is estimated by the ordinary least square (OLS) method, and then the estimated residuals are squared to estimate the following equation:

(A3) $${\hat{\sigma }}_{OLS,i}^{2}=\beta X_{i}+\theta_{i}$$

Secondly, the estimated ${\hat{\beta }}_{OLS}$ of this auxiliary regression is used to transform Equation (A3) as follows:

(A4) $$\frac{{\hat{\sigma }}_{OLS,i}^{2}}{{\hat{\beta }}_{OLS}X_{i}}=\beta \left(\frac{X_{i}}{{\hat{\beta }}_{OLS}X_{i}}\right)+\frac{\theta_{i}}{{\hat{\beta }}_{OLS}X_{i}}$$

Equation (A4) is estimated with OLS, which gives a consistent estimate,$\;{\hat{\beta }}_{FGLS}X_{i}$, of the variance of the idiosyncratic component of the food expenditure, $\sigma_{\mu ,i}^{2}$. Then, the standard error of ${\hat{\beta }}_{FGLS}$ can be calculated using Equation (A5), as follows:

(A5) $${\hat{\sigma }}_{\mu ,i}^{\;}=\sqrt{{\hat{\beta }}_{FGLS}X_{i}}$$

Lastly, Equation (7) is transformed with the standard error to estimate α, as follows:

(A6) $$\frac{lnE_{i}}{{\hat{\sigma }}_{\mu ,i}^{\;}}=\alpha \left(\frac{X_{i}}{{\hat{\sigma }}_{\mu ,i}^{\;}}\right)+\frac{\mu_{i}}{{\hat{\sigma }}_{\mu ,i}^{\;}}$$

An OLS estimation of Equation (A6) gives an asymptotically efficient estimate, ${\hat{\alpha }}_{FGLS}$, of α. Using ${\hat{\alpha }}_{FGLS}$ and ${\hat{\beta }}_{FGLS}$, the expected log of food expenditure and its variance could be estimated as follows:

(A7) $$E\left(ln{\hat{E}}_{i}|X_{i}\right)={\hat{\alpha }}_{FGLS}X_{i}\;\mathrm{and}$$

(A8) $$Var\left(ln{\hat{E}}_{i}|X_{i}\right)=\;{\hat{\beta }}_{FGLS}X_{i}$$

By assuming that the food expenditure is log normally distributed, we are able to estimate vulnerability to food poverty by applying Equation (A9), as follows:

(A9) $${\hat{V}}_{i}=\mathrm{Pr}\left(lnE_{i,t+\Delta }<lnZ|X_{i}\right)=\Phi \left(\frac{lnZ-{\hat{\alpha }}_{FGLS}X_{i}}{\sqrt{{\hat{\beta }}_{FGLS}X_{i}}}\right)$$

The value of ${\hat{V}}_{i}\;$(ranging from 0 to 1) measures the probability of individual i with characteristics of $X_{i}$ will be in food poverty at time t+Δ. With a vulnerability threshold of 0.5, we define that the individual is vulnerable to food poverty if ${\hat{V}}_{i}>0.5$. Replacing the dependent variable of the fuzzy RD design described in Equation (5) with vulnerability to food poverty, the effect of pension income on vulnerability is investigated.
